# Characterization, phylogeny, alternative splicing and expression of *Sox30 *gene

**DOI:** 10.1186/1471-2199-11-98

**Published:** 2010-12-11

**Authors:** Fei Han, Zhijian Wang, Fengrui Wu, Zhihao Liu, Baofeng Huang, Deshou Wang

**Affiliations:** 1 Key Laboratory of Freshwater Fish Reproduction and Development (Ministry of Education), Key Laboratory of Aquatic Science of Chongqing, School of Life Science, Southwest University, Chongqing 400715, PR China

## Abstract

**Background:**

Members of the Sox gene family isolated from both vertebrates and invertebrates have been proved to participate in a wide variety of developmental processes, including sex determination and differentiation. Among these members, *Sox30 *had been considered to exist only in mammals since its discovery, and its exact function remains unclear.

**Results:**

*Sox30 *cDNA was cloned from the Nile tilapia by RT-PCR and RACE. Screening of available genome and EST databases and phylogenetic analysis showed that *Sox30 *also exists in non-mammalian vertebrates and invertebrates, which was further supported by synteny analyses. Tissue expression in human, mouse and tilapia suggested that *Sox30 *was probably a gonad-specific gene, which was also supported by the fact that *Sox30 *EST sequences were obtained from gonads of the animal species. In addition, four alternatively spliced isoforms were isolated from tilapia gonad. Their temporal and spatial expression patterns during normal and sex reversed gonadal development were investigated by RT-PCR and *in situ *hybridization. Our data suggest that expressions of *Sox30 *isoforms are related to stage and phenotypic-sex, observed in the germ cells of male gonad and in somatic cells of the female gonad.

**Conclusions:**

*Sox30 *is not a gene only existed in mammals, but exists widely throughout the animal kingdom as supported by our bioinformatic, phylogenetic and syntenic analyses. It is very likely that *Sox30 *is expressed exclusively in gonads. Expression analyses revealed that *Sox30 *may be involved in female and male gonadal development at different stages by alternative splicing.

## Background

Since the discovery of *Sry *[1-3], numerous genes encoding proteins containing HMG (High Mobility Group) domain have been identified throughout the animal kingdom [4-6]. Genes with their HMG domains similar to that of *Sry *(testis determining gene in mammals) by at least 50% were named as *Sox *(SRY-related HMG-box) genes [5]. At present, *Sox *genes encoding transcription factors, count about 30 members in vertebrates [6,7]. Based on differing levels of structural, organizational similarities and other features, *Sox *genes can be subdivided into 10 groups, named from A to J [8]. Members of the same group are similar to each other within and outside the HMG-box. However, members from different groups share lower degree of identity in the HMG-box, and no significant identity outside this domain. Many of the Sox proteins, like Sry, Sox2, Sox3, Sox11 and Sox22, are encoded by a single exon [9-12]. In contrast, others, such as *Sox6*, *Sox9*, *Sox17 *and *Sox30*, consist of multiple exons [13-16]. In addition, the *Sox *genes are scattered on different chromosomes and are not clustered [5]. Apart from the *Sry *and *Sox3 *located on the Y- and X-chromosome respectively, other *Sox *genes are autosomal [17].

*Sox30 *(SRY-box containing gene 30), the only member of group H, has been characterized merely in a few species so far. It was firstly isolated from mouse (*Mus musculus*) and human (*Homo sapiens*). In these species, two transcripts of *Sox30 *mRNA were found, one of which encodes a truncated protein lacking partial C-terminal region due to a frame shift [16]. *Sox30 *was considered to be involved in mammalian spermatogonial differentiation and spermatogenesis [16,18]. As no sequences of *Sox30 *were available from any other groups of animals, including the sequenced fish genomes at that time, some scholars even speculated that *Sox30 *was specific to mammals [19]. In zebrafish (*Danio rerio*), a *Sox30 *gene has been reported [20], however, it was proved to be *Sox21 *by GenBank blast and phylogenetic analysis. Interestingly, in our present study, *Sox30 *was isolated from the Nile tilapia (*Oreochromis niloticus*) accidentally when cloning *Sox9b*. Subsequently, by blast search and data mining, *Sox30 *was also found to exist in genome sequences of the chicken (*Gallus gallus*), green anole lizard (*Anolis carolinensis*), western clawed frog (*Xenopus tropicalis*), sea squirts (*Ciona intestinalis*), lancelets (*Branchiotoma belcheri*), acorn worm (*Saccoglossus kowalevskii*), sea anemone (*Nematostella vectensis*), and ESTs (Expressed Sequence Tags) of the channel catfish (*Ictalurus punctatus*), guppy (*Poecilia reticulata*), fathead minnow (*Pimephales promelas*), little skate (*Leucoraja erinacea*), dogfish (*Squalus acanthias*), the California mussel (*Mytilus californianus*) and snail (*Lottia gigantean*). Thus we report here that *Sox30 *exists widely throughout the animal kingdom rather than specific to mammals as reported previously. More importantly, we isolated four alternatively spliced isoforms of *Sox30*, and studied their temporal and spatial expression patterns during normal and sex reversed gonadal development in tilapia. Our data suggest that *Sox30 *may play key roles in gonadal development, and the roles may be different at different stages and sexes.

## Methods

### Animals

The Nile tilapias for this study were reared in large tanks with a circulating aerated freshwater system. Fish were maintained at ambient temperature (26°C) under natural photoperiod. All genetic females (XX) and males (XY) were obtained by artificial fertilization of eggs from normal females (XX) with sperms from either sex reversed males (XX) or super males (YY), respectively [21]. Then, artificially fertilized eggs were cultured in recirculating water at 26°C to obtain fry. All animal experiments conformed to the Guide for Care and Use of Laboratory Animals and were approved by the Institutional Committee of Laboratory Animal Experimentation at Southwest University, China.

### Drug treatment

XX males and XY females were generated by treating fry with estrogen receptor antagonist tamoxifen (TAM) (Novartis Company, AG, Switzerland) and 17β-estradiol (E2) (Sigma, USA), respectively. Fish feed were sprayed with 95% ethanol containing TAM, 25 μg/g; E2, 50 μg/g. Control fish were fed with 95% ethanol sprayed feed. Drug treatment was applied to the fry from 5 to 30 days after hatching (dah), the critical period of the Nile tilapia sex differentiation [22]. E2 (XY) and TAM (XX) treatment resulted in complete sex reversal in tilapia, respectively [23].

### Cloning of tilapia *Sox30 *cDNA and genomic DNA

The SMART RACE cDNA of the gonads, which was used to perform both 5' and 3'-rapid implication of cDNA ends (RACE) to obtain full-length cDNA of tilapia *Sox30*, were synthesized according to the manufacturer's instruction (Clontech). The *Sox30 *5' cDNA end was amplified accidentally with primers for *Sox9b *(partial sequence 225 bp, accession number: DQ632575), and confirmed by blastx http://blast.ncbi.nlm.nih.gov/. Subsequently, 3' RACE primers were designed to amplify 3' cDNA end for tilapia *Sox30*. Finally, to verify the full-length sequences of *Sox30 *cDNA, end-to-end PCR was performed and four mRNA isoforms were identified by using 5'- and 3'-UTR primers (T30UTR-F, T30UTR-R). In order to determine the gene structure of the tilapia *Sox30*, the genomic DNA sequence of this gene was amplified. Genomic DNA was prepared from mature testis and ovary. A 3790 bp *Sox30 *gene fragment with complete cds (coding sequences) and four introns, 102, 1518, 106 and 980 bp in length, was obtained by genomic PCR using end primers T30UTR-F and T30UTR-R. All the primers and sequences used in the present study were listed in Table 1. The PCR products were resolved on a 1.2% agarose gel and the target DNA fragments were purified using QIAquick Gel Extraction Kit (Qiagen, Germany).

**Table 1:** Sequence of primers used in the present study

Primer	Sequence	Purpose
R1	GTAGTCCGGGTGATCCTTCTTGTGC	RACE
R2	AGACGCTCAGCCTCCTCCACGAA	RACE
R3	AGCCTGAGCCCACACCATGAACG	RACE
F1	TCAGCGTCAGCCCAGGTCAGGAGA	RACE
F2	TGTTTCAGGGCTGTGGAGTTGGTGG	RACE
F3	GCAAACGCAAACTGGCAGAGACGC	RACE
UTR-F	CGGTCTCACAATGGGAACCAACTC	Full-length cDNA amplification and tissue distribution
UTR-R	GCTGAACATCTCCTAGAGCAGTCCA	Full-length cDNA amplification and tissue distribution
β-actinF	GGCATCACACCTTCTACAACGA	Internal control
β-actinR	ACGCTCTGTCAGGATCTTCA	Internal control

All the fragments obtained were cloned into pGEM-T Vector (Promega, USA) and bi-directional sequencing was performed by the dideoxy chain termination method using an ABI PRISM 377 DNA genetic analyzer (Sangon, Shanghai, China).

### Data mining and sequence analyses

The following genome databases were searched to explore the presence of *Sox30*-like sequences in other vertebrate and invertebrate species, including the UCSC Genome Bioinformatics at: http://genome.ucsc.edu/, the NCBI databases at http://blast.ncbi.nlm.nih.gov/, the Silk DB databases at http://silkworm.genomics.org.cn/, the JGI databases at http://genome.jgi-psf.org/Nemve1/Nemve1.home.html, the Ensembl databases http://www.ensembl.org/biomart/martview/ and the Gene DB databases at http://www.genedb.org/genedb/smansoni/. *Sox30*-like sequences were identified from seven species by blast search against genome sequences and further confirmed in EST databases, i.e., chicken (XP_414564, CN227447, CN229457, CO771932 and BU460467), anole lizard (scaffold_69: 1510867-1526492), frog (scaffold_ 177: 129323-130299, CX934092 and CX934091), sea squirts (NM_001078355), lancelets (chrUn: 451615932-451618210, BW716506, BW714230, BW695368 and BW697885), acorn worm (scaffold_16907: 1180000-1220000) and sea anemones (scaffold_134:263216-265501), but failed to identify it from other 80 animal species with genome sequence information. Partial or complete *Sox30*-like sequences were also identified by blast search against the NCBI EST databases from channel catfish (FD337967), guppy (ES386169), fathead minnow (DT310961, DT345945, DT310040, DT100872 and DT266812), little skate (GH161005 and CV222391), dogfish (CX196372), the California mussel (ES397482) and snail (FC701316, FC674522, FC687599 and FC698472).

The orientation and chromosomal position of *Sox30 *and its adjacent genes *Thg1l *(tRNA-histidine guanylyltransferase 1-like) and *Adam19 *(ADAM metallopeptidase domain 19) were determined manually from the gene orientations listed in BIOMART from the Ensembl database. Synteny analyses of *Sox30*, *Thg1l *and *Adam19 *in human, mouse, chicken, anole lizard and frog were performed by comparing the cDNA sequences with genome sequence using BLAST search from the UCSC Genome Bioinformatics or by search in BIOMART from the Ensembl database.

The multiple alignment software DNAstar and ClustalX were employed to analyze the nucleotide sequences and their deduced amino acid (aa) sequences. Based on the alignment results, a phylogenetic tree of Sox30 proteins was constructed using the fruitfly (*Drosophila melanogaster*) SoxF as an outgroup. The *Sox30 *sequences isolated from chicken, anole lizard, frog, channel catfish, guppy, fathead minnow, little skate, dogfish, sea squirts, lancelets, acorn worm, the California mussel, snail and sea anemones were also included in the phylogenetic analysis. The tree was constructed using the neighbor-joining method. The scores represent bootstrap values of 1000 trials, indicating the credibility of each branch. All the aa sequences used in the phylogenetic analysis were obtained from GenBank, UCSC and JGI Genome Bioinformatics, except that from tilapia. The GenBank accession numbers and scaffold numbers of these sequences are listed in Table 2.

**Table 2:** Sequences used by this study

Name	Species	Group	**Accession No**.	**Pubmed No**.
SoxF (Sox15)	fruitfly, *Drosophila * melanogaster	F	NM_079015	10731132
Sox30	sea squirts,*Ciona intestinalis*	H	NM_001078355	15269171
Sox30*	lancelet, *Branchiostoma floridae*	H	chrUn:45161593 2-451618210	unpublished
Sox30*	western clawed frog, *Xenopus tropicalis*	H	scaffold_177:129323-130 299, CX934091 and CX934092	unpublished
Sox30	mouse, *Mus musculus*	H	NP_775560	16564520
Sox30	chicken, *Gallus gallus*	H	XP_414564	unpublished
Sox30	the Nile tilapia, *Oreochromis niloticus*	H	GQ463453	unpublished
Sox30	human, *Homo sapiens*	H	NP_848511	10359848
Sox30	cattle, *Bos taurus*	H	NM_001046429	19393038
Sox30*	mussel, *Mytilus californianus*	H	ES397482	unpublished
Sox30*	catfish, *Ictalurus punctatus*	H	FD337967	unpublished
Sox30*	guppy, *Poecilia reticulata*	H	ES386169	unpublished
Sox30	fathead minnow, *Pimephales promelas*	H	DT310961, DT345945 DT310040, DT100872 and DT266812	unpublished
Sox30*	snail, *Lottia gigantean*	H	FC701316, FC674522 FC687599 and FC698472	unpublished
Sox30	sea anemone, *Nematostella vectensis*	H	scaffold_134:2632 16-265501	unpublished
Sox30	acorn worm, *Saccoglossus kowalevskii*	H	scaffold_134:1180000 -1220000	unpublished
Sox30*	little skate, *Leucoraja erinacea*	H	GH161005 and CV222391	unpublished
Sox30*	dogfish, *Squalus acanthias*	H	CX196372	unpublished
Sox30	anole lizard, *Anolis carolinensis*	H	scaffold_69: 1510867-1526492	unpublished

Note. * Partial sequences identified from databases and first named in this report.

### Analysis of *Sox30 *expression by RT-PCR and semiquantitative RT-PCR

Total RNAs (2.0 μg) were isolated from various tissues of adults (240dah) (XX and XY fish, for tissue distribution analysis) and the gonads of monosex (XX and XY fish at 5, 10, 15, 35, 90, 150, 240dah, for ontogeny analysis). Thereafter, total RNAs were treated with DNase I to eliminate the genomic DNA contamination. Then first strand cDNAs were synthesized and RT-PCR was carried out to check the expression levels of tilapia *Sox30 *according to methods described previously [24]. The templates for positive and negative controls were set with *Sox30 *plasmid DNA and deionized water, respectively. A 342-bp fragmentofβ-actin was amplified as internal control to test the quality of the cDNAs used in the PCR. The PCR products were subjected to agarose gel (1.2%) electrophoresis.

To examine the influence of drug treatment on *Sox30 *gene expression, gonads of 5 individuals were collected from each control group (XX female, XY male) and drug treated group (TAM-induced XX male and E2-induced XY female) when the fish were adult at 240dah. Semi-quantitative RT-PCR [25] was performed to measure the mRNA levels using the primers (T30UTR-F, T30UTR-R) described in the previous section. A series of PCRs with different cycle numbers (from 22 to 36, with an interval of 2) were performed to determine the linear phase of the amplification using 1 to 5 diluted cDNA template. Based on these pilot experiments, 28 cycles for β-actin and 34 cycles for the target gene were chosen and applied to the subsequent semi-quantitative RT-PCR analyses. Band intensities resulting from the PCR amplification were analyzed using the image analysis software Quantity One (Bio-Rad). *Sox30 *alternatively spliced mRNA levels were expressed relative to that of β-actin in each sample. In our study, β-actin expression was found to be unaffected by the drug treatment [23]. Results obtained were expressed as mean values ± S.E.M. from five individual fish. Data analyses were performed using one-way ANOVA and the least significant difference on the GraphPad Prism 4 software (GraphPad Software, San Diego, CA, USA).

### *In situ *hybridization (ISH)

Tilapia gonads from 10, 120 and 210dah were dissected and fixed in 4% paraformaldehyde (PFA) in 0.85× PBS (pH 7.4) at 4°C overnight. After fixation, gonads were embedded in paraffin. Cross-sections of 5 μm were cut with a sliding microtome. Probes of both sense and antisense digoxigenin (DIG)-labeled RNA strands were transcribed *in vitro *from a linearized tilapia *Sox30 *plasmid, using the RNA labeling kit (Roche Diagnostics GmbH, Mannheim, Germany). *In situ *hybridization was carried out as follows: sections were deparaffinized, hydrated and treated with proteinase K (10 μg/ml, Amersco, USA) and then hybridized with the sense or antisense DIG-labeled RNA probe at 60°C for 16-22 h. The hybridization signals were then detected using alkaline phosphatase-conjugated anti-DIG antibody (Roche, Germany) and NBT as the chromogen [26].

## Results

### Molecular cloning of tilapia *Sox30*

**Figure 1:**
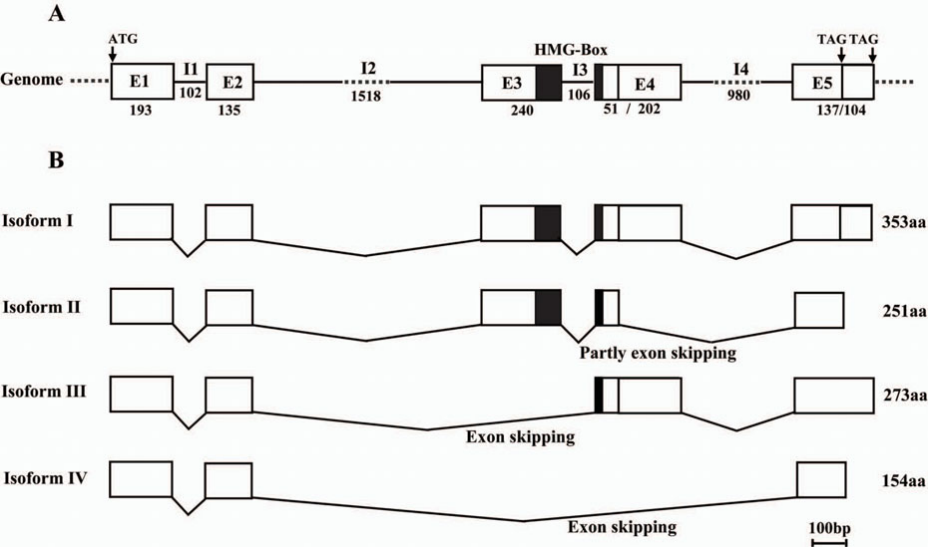
**Tilapia *Sox30 *gene structure (A) and its four alternatively spliced isoforms (B)**. Rectangle, solid line and dotted line represent exon, intron and omitted sequences, respectively. Numbers represent the lengths of nucleotide and protein sequences. E and I stand for exon and intron, respectively. ATG and TAG are start and stop codon, respectively. The HMG-box is shaded.

The *Sox30 *genomic DNA and four alternatively spliced cDNAs were cloned from the Nile tilapia. The genomic DNA is 3790 bp long (GenBank accession no. GQ463457), containing 5 exons, 193, 135, 240, 253 and 241 bp, respectively, and four introns (two in the N-terminal region, one in the C-terminal region and one within the HMG-box). All introns share the typical intron characteristics (gt----ag). The isolated full-length *Sox30 *cDNA (isoform-I) is 1474 bp (GenBank accession no. GQ463453), containing an open reading frame (ORF) of 1062 bp encoding a putative protein of 353 aa, a 116 bp 5' untranslated region (UTR), and a 296 bp 3' UTR. The other three alternatively spliced isoforms (isoform-II, -III and -IV, GenBank accession nos. GQ463454, GQ463455 and GQ463456) amplified by the same pair of primers were 306, 240 and 597 bp shorter than the ORF of isoform-I due to exon skipping. The four isoforms share the same 5'-UTR, while only isoform-I and -III share the same 3'-UTR. Isoform-II and -IV have 3'-UTRs 104 bp longer than those of isoform-I and -III due to the reading frame shift and introduction of a stop codon which is 104 bp upstream of the stop codon found in isoform-I and -III transcripts. Further analysis suggested that these isoforms (I-IV) contain 5, 5, 4 and 3 exons, respectively. Compared with the longest isoform (isoform-I, which contains 5 exons), part of exon 4, exon 3, exon 3 and exon 4 was spliced off in Isoform-II (with an ORF of 756 bp encoding 251aa), -III (822 bp encoding 273aa), and -IV (465 bp encoding 154aa), respectively (Figure 1).

### Sequence, phylogeney and synteny analyses

**Figure 2:**
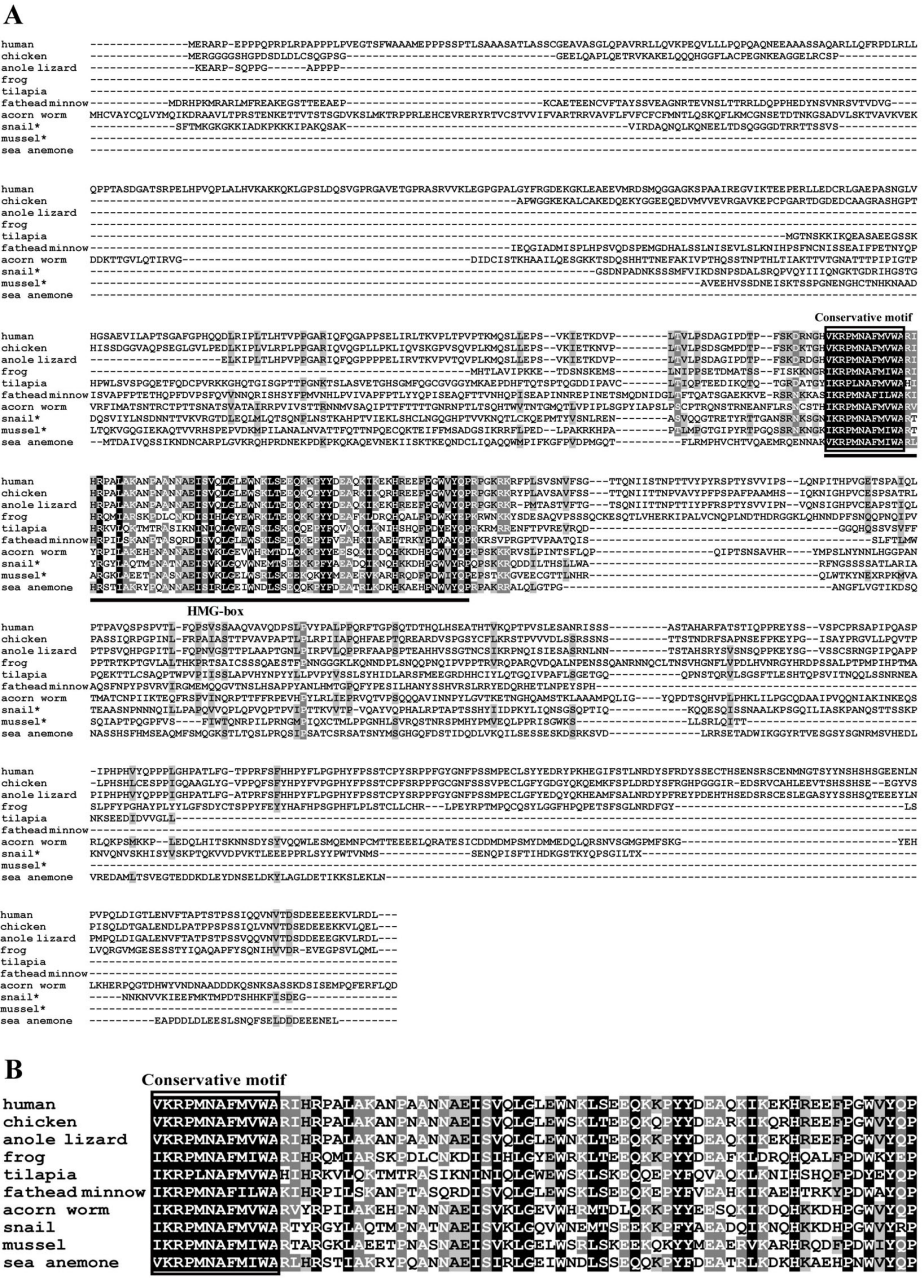
**Alignment of the amino acid sequences of Sox30 s (A) and their HMG-boxes (B)**. The HMG-box is underlined. The conservative motif in HMG-box is boxed. * indicates partial sequences.

**Figure 3:**
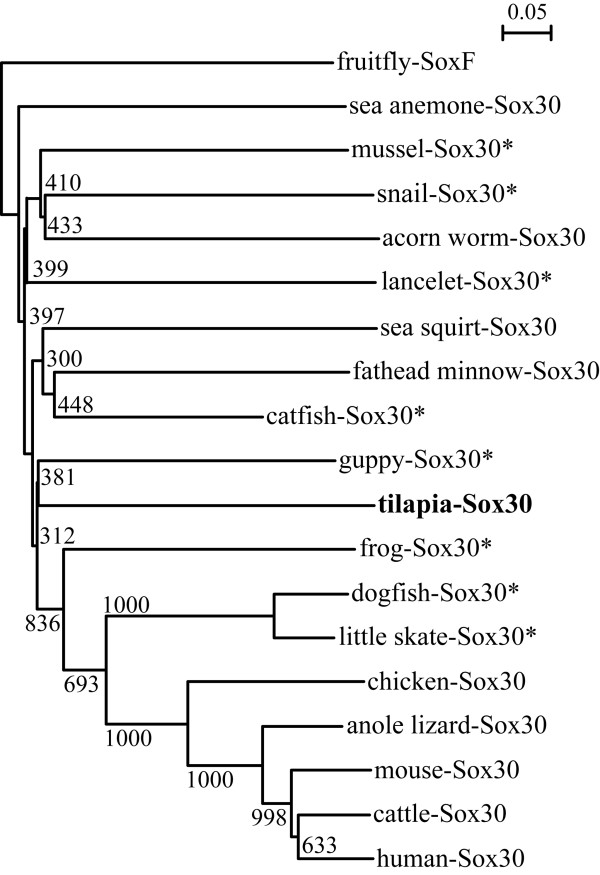
**Phylogenetic analysis of Sox30 s based on alignment of their full-length amino acid sequences**. This tree was generated by the default settings of Clustal × alignment program and constructed using fruitfly SoxF as an outgroup. The values in the tree represent bootstrap scores out of 1000 trials, indicating the credibility of each branch. Branch lengths are proportional to the number of amino acid changes on the branch. * indicates partial sequences. The little skate and dogfish Sox30 s were clustered in a wrong position because the isolated two sequences are very short, including only partial N- terminal and HMG-box regions.

**Figure 4:**
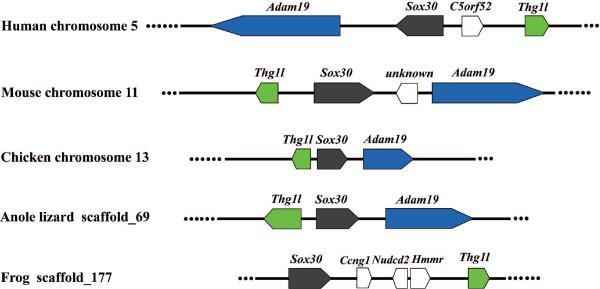
**Synteny analyses of *Sox30 *and its adjacent gene *Thg1l *and *Adam19 *in human, mouse, chicken, anole lizard and frog**. Rectangles represent genes in chromosome/scaffold, dotted lines represent omitted genes of the chromosome/scaffold, and the arrow represents gene-coding direction. *Sox30*, *Thg1l *and *Adam19 *are shown in grey, green and blue, respectively. The other genes are shown in white.

Multiple alignment of the aa sequences of Sox30 s from various species revealed that Sox30 was poorly conserved except in the HMG-box, the characteristic DNA binding domain (Figure 2). The HMG-boxes of tilapia and guppy Sox30 shared the highest similarity (about 52.9%), whereas the whole peptides of both shared rather low similarity (about 28%). Compared with Sox30 from mammals, tilapia Sox30 is shorter in both N- and C-terminal regions (Figure 2A). As was shown in Figure 3, Sox30 s isolated from tilapia and other species were clustered into the same clade with the mammalian Sox30 s, indicating that they are genuine Sox30 s. Synteny analyses showed that *Sox30 *and its adjacent gene *Thg1l *and *Adam19 *were located on the same chromosome/scaffold in the same gene order in human, mouse, chicken, anole lizard and frog (Figure 4).

### Tissue expression of *Sox30 *gene

**Figure 5:**
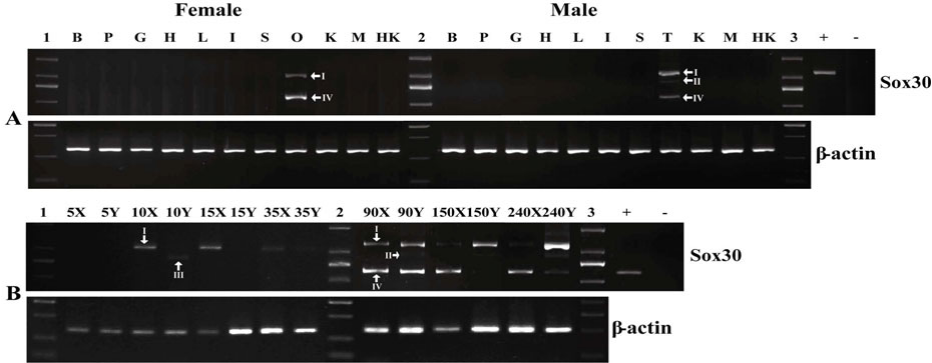
**Expression patterns of *Sox30 *in the Nile tilapia**. (A) RT-PCR analysis of *Sox30 *from various tissues of adult fish. B, brain; P, pituitary; G, gill; H, heart; L, liver; I, intestine; S, spleen; O, ovary; K, kidney; M, muscle; HK, Head kidney; T, testis; 1, 2, and 3, marker; +, positive control; -, negative control; β-actin was used as the internal control; (B) Ontogeny (5-240 dah) of *Sox30 *expression in tilapia gonads by RT-PCR. 1, 2, marker; X, female; and Y, male; +, positive control; -, negative control; I, II, III, IV indicate isoform-I, -II, -III and -IV, respectively.

Tissue distribution analysis using RT-PCR revealed that only three of the four isoforms of tilapia *Sox30 *(isoform-I, -II and -IV) were expressed in adult. They were expressed exclusively in gonads, with isoform-I higher in the testis; isoform-IV higher in the ovary; while isoform-II only in the testis, not in the ovary (Figure 5A).

### Ontogeny of *Sox30 *expression in tilapia gonads

*Sox30 *expression was detected in gonads of tilapia during sex determination, differentiation and later development by RT-PCR. Isoform-I mRNA was detected consistently in the gonads at 10, 15, 35, 90, 150 and 240 dah with a very interesting expression profile: ovary only at 10 and 15 dah; both ovary and testis from 35 dah onwards, higher in testis than in ovary from 90 dah to adult (Figure 5B). Isoform-II showed a male-specific expression from 90 dah to adult. Isoform-III was detected exclusively in male gonad at 10 dah only, and no expression was detected during any other developmental stages. Isoform-IV started to express in both testis and ovary from 90 dah onwards, with higher level in the ovary from 150 dah to adult.

### Expression of *Sox30 *in sex-reversed gonads

**Figure 6:**
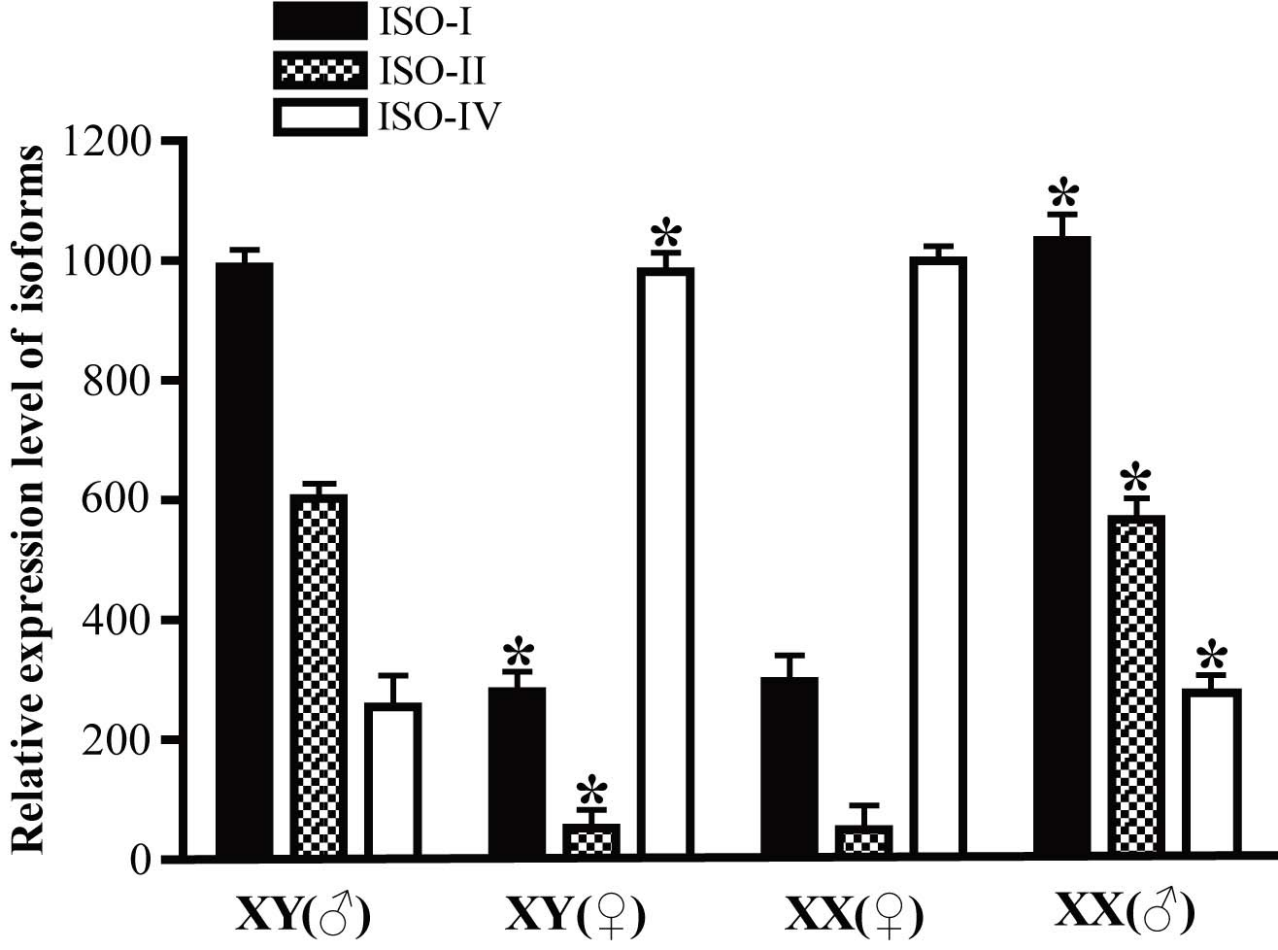
**Relative expression levels of tilapia *Sox30 *isoforms in gonad of control and sex reversed adult tilapia by semi-quantitative RT-PCR**. Results were expressed as mean values ± S.E.M. from five individual fish (*P < 0.01 significantly different as compared with the respective control by one-way ANOVA). Iso is the abbreviation of isoform. XX (♂), XX male; XY (♀), XY female; XY (♀) fish, male-to-female sex reversal induced by 17β-estradiol (E2); XX (♂) fish, female-to-male sex reversal induced by tamoxifen (TAM). The doses of the drugs used are described in the Materials and Methods section.

Consistent with tissue distribution results, three isoforms (I, II and IV but not III) were detected in adult gonads by semi-quantitative RT-PCR. Isoform-I and -IV mRNA were detected in gonads of all four groups, i.e. XX (♀) and XY (♂) control fish and sex reversed XX (♂, induced by TAM treatment) fish and sex reversed XY (♀, induced by E2 treatment) fish. Isoform-I was significantly down-regulated in sex reversed XY (♀) gonads, but up-regulated in sex reversed XX (♂) gonads, being consistent with the result of tissue distribution and ontogeny analyses that isoform-I was expressed higher in the testis; Isoform-IV was up-regulated in sex reversed XY (♀) gonads, but down-regulated in sex reversed XX (♂) gonads, which was consistent with that isoform-IV was expressed higher in the ovary. Isoform-II mRNA was only detected in normal XY (♂) and sex reversed XX (♂) gonads but not in normal XX (♀) and sex reversed XY (♀) gonads, consistent with that isoform-II showed a male gonad-specific expression in tissue distribution and ontogeny analyses. These data indicated that the expressions of *Sox30 *are phenotypically related rather than genotypically related (Figure 6).

### Expression of *Sox30 *by *in situ *hybridization in tilapia gonads

**Figure 7:**
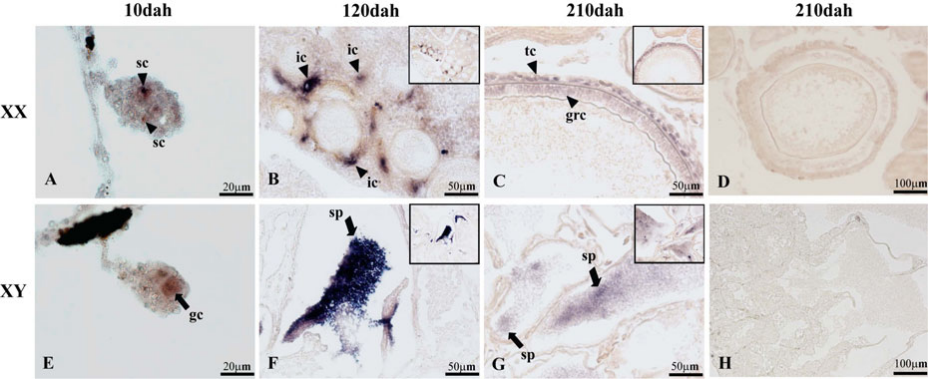
**Expression of tilapia *Sox30 *in gonads examined by *in situ *hybridization with antisense (A-C, E-G) and sense (D, H) probes**. At 10 dah, *Sox30 *expression appears in germ cells (gc) of the male gonad (E), and in a few round somatic cells (sc) of the female gonad (A); at 120 dah, *Sox30 *was expressed in sperms (sp) of the testis (F) and interstitial cells (ic) of the ovary (B); at 210 dah, *Sox30 *was expressed in sperms of the testis (G), theca (tc) and granulosa cells (grc) of the ovary (C). The arrow and arrowhead indicate signals of *Sox30 *in the XY and XX gonads, respectively.

To ascertain which population of cells in the developing gonads expresses *Sox30*, ISH was performed using gonads at 5, 10, 120 and 210 dah. Specific signals were observed in the male (XY) germ cells at 10 dah (Figure 7E) and the sperms of the testis at 120 and 210 dah (Figure 7F, G). In contrast, specific signals were detected in the female (XX) somatic cells at 10 dah (Figure 7A), interstitial cells of the ovary at 120 dah (Figure 7B), and theca and granulosa cells of the ovary at 210 dah (Figure 7C), but no signal was detected in both sexes at 5 dah (data not shown). Taking the ontogeny results into consideration, we conclude that isoform-III is restricted to the germ cells in male gonads and isoform-I is expressed in the somatic cells of the female gonads at 10 dah.

However, due to the overlapping probes (The probes used in this study recognize all the four *Sox30 *isoforms. It is impossible to design isoform-specific probe for all of them), we failed to distinguish the signals detected at 120 and 210 dah gonads. At least three and two isoforms could produce the signals detected in the testis and the ovary, respectively at these stages.

## Discussion

### Bioinformatics and phylogeney analyses of *Sox30*

In the present study, a novel *Sry*-related gene was isolated from the Nile tilapia accidentally when cloning *Sox9b*. It was characterized as *Sox30 *by subsequent blast against GenBank for its relatively high identity with its mammalian orthologs, which was further confirmed by phylogenetic and syntenic analyses. To date, *Sox30 *has been reported only in mammals [16,18,19], but not in any non-mammalian vertebrates (including fish) and invertebrates. Our data, for the first time, provided the solid proofs for the existence of *Sox30 *in a teleost fish, the Nile tilapia.

Subsequently, genome databases of fishes, including zebrafish, medaka (*Oryzias latipes*), stickleback (*Gasterosteus aculeatus*), takifugu (*Takifugu rubripes*) and tetraodon (*Tetraodon nigroviridis*) were searched for possible *Sox30*-like genes. However, it seems that this gene does not exist in any database of these teleosts. EST data bases were also searched afterward, and complete or partial *Sox30 *sequences were successfully isolated from the channel catfish, guppy, fathead minnow, little skate and dogfish. Further screening of the genome sequences of other animals showed that *Sox30 *was also present in other chordates, including chicken, anole lizard, frog, sea squirts, lancelets and acorn worm (also called as *SoxH *in the last three species), and non-chordate invertebrates, including the California mussel, snail and sea anemone (also called as *SoxH*), while not found in the nematode (*Caenorhabditis elegans*), fruitfly and sea lamprey. A recent report stated the existence of *Sox30*-like sequence in sea urchin (*Strongylocentrotus purpuratus*) [27], but we failed to find in the genome and EST databases by blast search. These findings suggested that *Sox30 *is not specific to mammals, but also exists in other vertebrates and invertebrates. Then, why we failed to find *Sox30*-like sequences in all sequenced teleost genomes? There are two possible explanations. One is that the genome sequences from these species have not covered all the genome yet; and the other is that *Sox30 *was secondarily lost in these species during evolution. However, the chance for the latter possibility is quite small because of the following two reasons, 1) cyprinids and ictalurids, such as fathead minnow and channel catfish, are relatively primitive teleosts while cichlids and poeciliids, such as tilapia and guppy, are relatively advanced teleosts. It is unlikely that *Sox30 *was lost in all the other teleosts except the primitive and advanced species; 2) both fathead minnow and zebrafish belong to the same family, Cyprinidae. It is difficult to believe that *Sox30 *was secondarily lost in the zebrafish but not in another closely related species, fathead minnow. Therefore, we tend to accept the former explanation. To confirm this, further cloning *Sox30 *from other teleosts is required.

*Sox30 *was found not only in chordates such as human, mouse, chicken, frog, tilapia, channel catfish, guppy, fathead minnow, little skate, dogfish, sea squirts, lancelets and acorn worm, but also in non-chordate invertebrate species, such as the California mussel, snail and sea anemone, indicating that it had already appeared before or with the emergence of coelenterates. Then, it is of interests to know from which ancestor gene *Sox30 *was derived. Group H, which only consists of *Sox30*, shared the highest similarity to group F in 10 Sox groups (Additional file 1, Fig.S1), suggesting that they may share a common ancestor. However, the exact timing for the divergence of the two groups is still unclear. Its presence in the relatively primitive invertebrate species, sea anemones (no *Sox30 *or other *Sox *genes have been found in protozoa and bacteria at present) and its position (the outermost clade) in the phylogenetic tree (Additional file 1, Fig. S1) suggested that *Sox30 *seemed to be one of the oldest members in the Sox family, which is consistent with the previous conclusion [28].

### Sequence and gene structure analyses of *Sox30*

Compared with its counterparts from mammals, tilapia *Sox30 *genomic sequence was smaller in size due to compression of intron sequences. Similar phenomenon has been found for *Sox30 *in sea squirts, lancelets and acorn worm. Alignment of aa sequences indicated that Sox30 s was poorly conserved, even in the highly conserved HMG-box among both distant and closely related species, suggesting high diversity and rapid evolution of these proteins. This also explains why *Sox30 *has not been successfully isolated from non-mammalian animals until the accident cloning of tilapia *Sox30 *in this study. Gene structure analysis showed that tilapia *Sox30*, like some other Sox group members, such as *Sox8*, *Sox9 *and *Sox10 *[29], was also characterized by the splitting of the HMG-box by an intron. The same is true to *Sox30 *of acorn worm, lancelets, sea squirts, frog, chicken, mouse and human, and the location of the intron is conserved among these species. These findings indicated that this intron had already been fixed before or with the emergence of chordates.

Alternative splicing (AS) is emerging as one of the most important mechanisms to control vertebrate gene expression. Existing data indicate that as much as 76% of genes generate alternatively spliced products [30]. AS has been associated with a regulatory system in tissue- or stage-specific splicing mechanisms by which expression may be regulated. This regulation may be achieved by the introduction of premature stop codons that function as an on-off gene expression switch [31]. Previous study showed that two different mRNA isoforms of *Sox17*, *Sox30 *have been isolated in mouse and two different mRNA isoforms of *Sox9 *in frog because of AS [15,16,32]. In mice, the *Sox17 *isoform is expressed at a low level in the testis throughout postnatal development, while the t-*Sox17 *isoform is expressed abundantly in the testis, predominantly in postmeiotic germ cells [15]. Based on these results, they suggested that a switch from *Sox17 *to the t-*Sox17 *isoform may alter the function of *Sox17 *at the meiotic and postmeiotic phases during spermatogenesis in mice [15]. Our data demonstrated that tilapia *Sox30 *has four alternatively spliced isoforms. Early termination of the stop codon was found in the isoform-II and -IV transcripts of tilapia *Sox30*, suggesting that the mechanism mentioned above may be also responsible for regulation of *Sox30 *gene expression in tilapia. Besides, similar to mouse *Sox17*, four isoforms of *Sox30 *were also expressed in a non-parallel manner during gonadal development of tilapia. Thus, there may be some switches from one to the other isoform(s) to alter the function of *Sox30 *during gonadal development. It is worthwhile to note that the HMG-box region was deleted partially in isoform-III and completely in isoform-IV, respectively, which resulted in truncated proteins lacking most parts or all of the HMG-box domain and the nuclear localization signal.

It is interesting to know whether these protein isoforms are ever made, but now there is no antibody available because Sox30 is poorly conserved among species of different classes of animals, and therefore, the available antibody against mammalian Sox30 is not suitable for usage in fish. There are reports showing that alternatively spliced isoforms of *Sox17 *and *Sox30*, without functional DNA binding domain or C-terminus, very similar to tilapia *Sox30 *isoforms, were translated into proteins in mammals [15,16]. Based on those findings, we speculated that all isoforms of *Sox30 *in tilapia may also be translated into the protein products. As *Sox30 *gene, like *Sry *and some other *Sox *genes, uses the single HMG-box for DNA-binding, it would be very interesting to know what these truncated isoforms do and how they function. Whether this would result in different capacity of transcription activation, e.g. complete non-functional factors or dominant negative mutants of the wild type Sox30, is still an open question.

### The expression pattern and functional relevance of *Sox30 *in tilapia

Previous reports in mammals showed that human and mouse *Sox30 *are exclusively expressed in normal adult testis and specifically in germ cells [16]. This expression pattern suggests that *Sox30 *may be involved in mammalian spermatogonial differentiation and spermatogenesis [18] However, it is still unclear in which cell type *Sox30 *is expressed in the mammalian ovary even though there was report indicating its possible expression in mouse oocytes [33]. In the present study, *Sox30 *started to express in gonads from 10 dah, earlier than the morphological gonadal differentiation period (about 25 dah) in tilapia and showed a gonad specific expression pattern at least in adults. Of course, expression of *Sox30 *in extra-gonadal tissues in other stages of development cannot be completely excluded. Meanwhile, expression of each alternatively spliced isoform showed a clear sexual dimorphism in gonads. However, none of the four isoforms of *Sox30 *were detected at 5 dah, the critical period of tilapia molecular sex determination, which excluded its role as the sex determining gene in tilapia. In sex reversed adult gonads, three types of *Sox30 *also exhibited a phenotypic sex-related expression pattern. *Sox30 *expression was restricted to the germ cells at 10 dah and later to sperms in male gonads, indicating its possible involvement in spermatogonial differentiation and spermatogenesis in male fish, like in human and mouse. In female, it was expressed in the somatic cells in female gonads at 10, 120 and 210 dah, respectively.

*Sox30 *is a strong regulator of the *Sf-1 *[33], the most important steroidogenic factor found in all vertebrates, including fish [34,35]. Our in situ hybridization data demonstrated clearly that *Sox30 *is expressed in the somatic cells, especially steroidogenic cells of the ovary, colocalized with *Sf-1 *in tilapia [34]. Taken together, we speculate that *Sox30 *may be an important regulator for somatic differentiation and steroidogenesis in female fish as well. Both isoform I and IV are expressed in the ovary after 35 dah. Although isoform IV is expressed much higher than isoform I it lacks the DNA binding HMG-box, and therefore, it is unlikely that this isoform regulates *Sf-1 *expression by direct binding to the promoter. However, the possibility that isoform IV might function as a dominant negative mutant of the wild type molecule (isoform I) can not be excluded because it still has the other functional domains, such as transactivation domain. More works need to be done to unravel this.

In addition to the gonad specific expression of *Sox30 *in human, mouse and tilapia, all *Sox30 *EST sequences were also derived from gonads (testis and ovary in chicken and channel catfish, testis in frog, guppy and fathead minnow, male gonads in snail) demonstrating that *Sox30 *may also be a gonad specific gene in these species. These data further supported that *Sox30 *plays a key role in gonadal differentiation and development, which might be conserved among species in the animal kingdom. Moreover, four alternatively spliced *Sox30 *isoforms exhibited different temporal and spatial expression patterns in tilapia gonads. Alternative splicing of *Sox30 *mRNAs and different temporal expression pattern of the spliced isoforms had also been reported in human and mouse [16]. Therefore, we speculate that *Sox30 *may be involved in gonadal differentiation and development in different sexes, at different stages and in different cell types of gonads in the animal kingdom by AS. Further study on *Sox30 *in other species is required to confirm this.

The low similarity between tilapia *Sox30 *and its mammalian counterpart raises a question of whether tilapia *Sox30 *is a genuine *Sox30 *or just a new Sox member of the group H. To answer this, we analyzed the synteny of *Sox30 *and its adjacent genes in human, mouse, chicken, anole lizard and frog (tilapia was not included because its genome sequences has not finished and open yet). *Sox30 *and its adjacent gene *Thg1l *and *Adam19 *were found to be located on the chromosome 5, 11, 13 and scaffold_69 and _177 in human, mouse, chicken, anole lizard and frog, respectively. The conservation in synteny between non-mammalian species and mammals indirectly support that our isolated *Sox30 s *are genuine orthologs of mammalian *Sox30*.

## Conclusion

For the first time, *Sox30 *was found to exist in non-mammalian vertebrates and invertebrates by cloning, genome and EST database analyses. The probable gonad specific expression in all species analyzed and the temporal and spatial expression of the alternatively spliced isoforms indicated that *Sox30 *may play a key role in gonadal differentiation and development, and the function of *Sox30 *may be regulated by AS and expression in different gender, different stages and different cell types in gonads in a wide variety of animals.

## Authors' contributions

FH carried out most of experiments, data mining and drafted the manuscript. ZJW co-carried out most of experiments and co-draft the manuscript. FRW participated in ISH. ZHL participated in some of expression study. BFH participated in gene cloning. DSW designed and supervised the experiment, analyzed data and critically edited the manuscript. All authors read and approved the final manuscript.
